# Supplementary material for: Reporting radiographers’ interaction with Artificial Intelligence—How do different forms of AI feedback impact trust and decision switching?
Source: PLOS Digit Health. 2024 Aug 7;3(8):e0000560. doi: 10.1371/journal.pdig.0000560 (PMC11305567; doi:10.1371/journal.pdig.0000560)
Supplement: S1 File — (DOCX) [file pdig.0000560.s001.docx]

**S1 File:**

Study transcript – example image set from examination

1. Do you think there are any pathology/ies visible on this radiographic image?

**Yes/no**

   
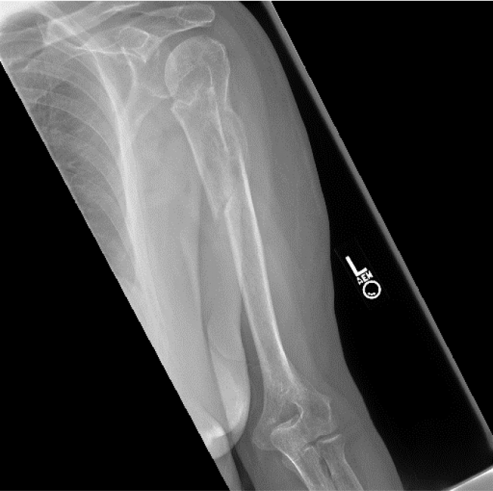


2. Please identify region(s) where any pathology is located, if applicable. If you believe there to be no pathology evident, please select ***no pathology.***

**Region(s) …..**


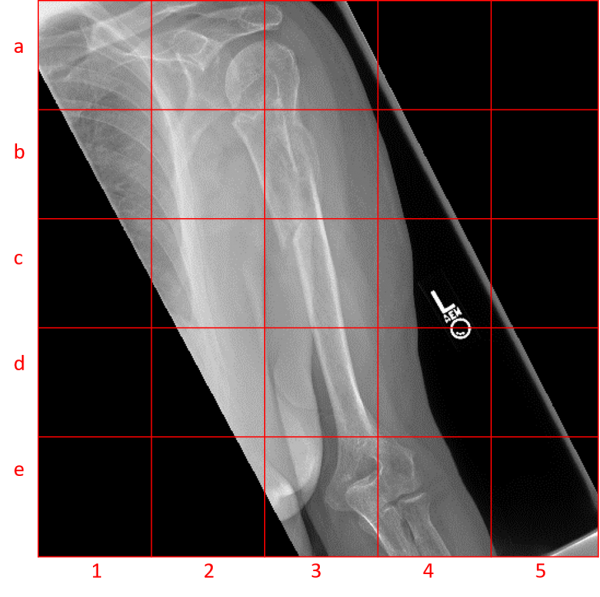


3. This is a heatmap produced by the AI model.  The WHITE area represents the area the AI system found ***most important*** in determining its diagnosis.  Does the area you found any abnormality match the white area on this image?  If you believe there to be no pathology evident, please select ***no pathology.***

**1. No pathology, 2. Yes -** in all the areas I have previously identified, **3. Yes -** in greater than half of the areas I have previously identified**, 4. Yes** - in less than half of the areas I have previously identified**, 5. Yes -** but there are also areas identified which I DO NOT consider to indicate pathology **, 6. No -** there are no areas of agreement with the area I have previously identified


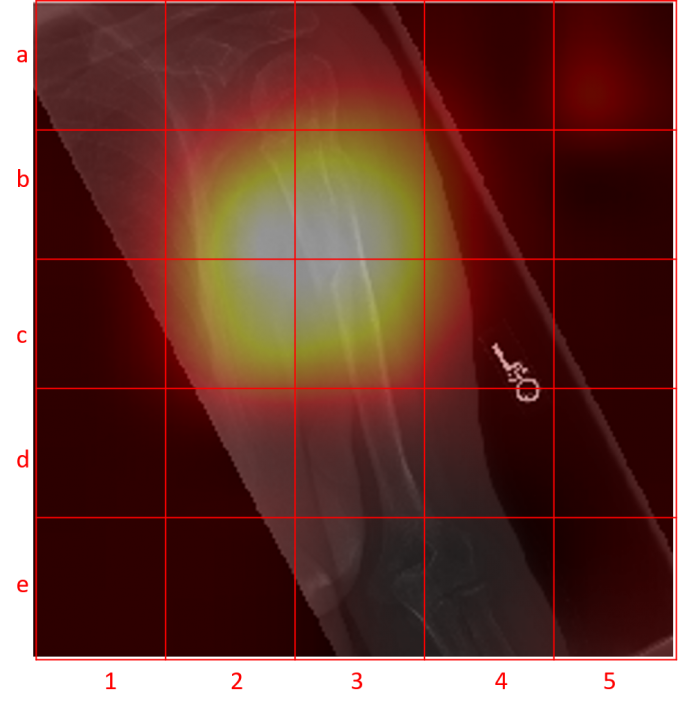


4. Following exposure to the AI heatmap, how would you rate your level of TRUST in the AI system, with 0 indicating no trust at all and 5 indicating absolute trust?

**0-5**

5. All images from this examination are given again below.  The AI system determined that this examination/imaging series DID contain evidence of pathology.  Do your initial diagnoses agree with this?

**Yes** - I think there is evidence of pathology on every image in the imaging series, **No** - I think that there is no evidence of pathology on any of the images in the imaging series, **Partly** - I think that some, but not all, images in the imaging series contained evidence of pathology

6. Following exposure to both the AI heat map and the AI diagnosis, how would you rate your level of TRUST in the AI system now, with 0 indicating no trust at all and 5 indicating absolute trust?

**0-5**

7. If you were presented with the heatmap and decision made by the AI ***for this examination*,**do you feel this would have caused you to change your mind about your initial diagnosis?

**Yes, maybe, no**

8. How would you rate the ***diagnostic quality*** of the images used in this examination, with 0 representing totally undiagnostic and 5 representing excellent diagnostic quality?

|  | 0 | 1 | 2 | 3 | 4 | 5 |
| --- | --- | --- | --- | --- | --- | --- |

| Diagnostic Quality perception Patient 1a () | 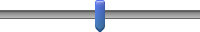 |
| --- | --- |

**This is the last question relating to this imaging series/examination.  Please click the arrow in the bottom right of the screen to progress to the next imaging series/examination.**
