# Supplementary material for: Reporting radiographers’ interaction with Artificial Intelligence—How do different forms of AI feedback impact trust and decision switching?
Source: PLOS Digit Health. 2024 Aug 7;3(8):e0000560. doi: 10.1371/journal.pdig.0000560 (PMC11305567; doi:10.1371/journal.pdig.0000560)
Supplement: S2 File — (DOCX) [file pdig.0000560.s002.docx]

**S2 File**: Per-participant diagnosis/examination and agreement with AI diagnosis

| **Image** |  | **n=** | **Pathology Y** | **Pathology N** | **TOTAL** | **EXPERT DIAGNOSIS - consensus** | **AI DIAGNOSIS** | **Expert/AI AGREE** | **EXPLAINATION** |
| --- | --- | --- | --- | --- | --- | --- | --- | --- | --- |
| 1a_1 |  | 5 | 5 | 0 | 10Y | 1 | 1 | Y |  |
| 1a_2 |  | 5 | 5 | 0 |  |  |  |  |  |
| 2a_1 |  | 5 | 1 | 4 | 6Y, 9N | 0 | 0 | Y |  |
| 2a_2 |  | 5 | 3 | 2 |  |  |  |  |  |
| 2a_3 |  | 5 | 2 | 3 |  |  |  |  |  |
| 3a_1 |  | 5 | 4 | 1 | 7Y, 3N | 1 | 0 | N | AI false neg |
| 3a_2 |  | 5 | 3 | 2 |  |  |  |  |  |
| 4a_1 |  | 5 | 0 | 5 | 1Y, 14N | 0 | 0 | Y |  |
| 4a_2 |  | 5 | 1 | 4 |  |  |  |  |  |
| 4a_3 |  | 5 | 0 | 5 |  |  |  |  |  |
| 5a_1 |  | 5 | 1 | 4 | 3Y, 12N | 0 | 1 | N | AI false pos |
| 5a_2 |  | 5 | 0 | 5 |  |  |  |  |  |
| 5a_3 |  | 5 | 2 | 3 |  |  |  |  |  |
| 6a_1 |  | 5 | 5 | 0 | 15Y | 1 | 1 | Y |  |
| 6a_2 |  | 5 | 5 | 0 |  |  |  |  |  |
| 6a_3 |  | 5 | 5 | 0 |  |  |  |  |  |
| 7a_1 |  | 5 | 0 | 5 | 1Y, 14N | 0 | 0 | Y |  |
| 7a_2 |  | 5 | 1 | 4 |  |  |  |  |  |
| 7a_3 |  | 5 | 0 | 5 |  |  |  |  |  |
| 8a_1 |  | 5 | 5 | 0 | 15Y | 1 | 1 | Y |  |
| 8a_2 |  | 5 | 5 | 0 |  |  |  |  |  |
| 8a_3 |  | 5 | 5 | 0 |  |  |  |  |  |
| 9a_1 |  | 5 | 0 | 5 | 2Y, 13N | 0 | 0 | Y |  |
| 9a_2 |  | 5 | 2 | 3 |  |  |  |  |  |
| 9a_3 |  | 5 | 0 | 5 |  |  |  |  |  |
| 10a_1 |  | 5 | 2 | 3 | 5Y, 10N | 0 | 0 | Y |  |
| 10a_2 |  | 5 | 0 | 5 |  |  |  |  |  |
| 10a_3 |  | 5 | 3 | 2 |  |  |  |  |  |
| 11a_1 |  | 5 | 0 | 5 | 2Y, 13N | 0 | 0 | Y |  |
| 11a_2 |  | 5 | 2 | 3 |  |  |  |  |  |
| 11a_3 |  | 5 | 0 | 5 |  |  |  |  |  |
| 12a_1 |  | 5 | 0 | 5 | 15N | 0 | 0 | Y |  |
| 12a_2 |  | 5 | 0 | 5 |  |  |  |  |  |
| 12a_3 |  | 5 | 0 | 5 |  |  |  |  |  |
| 1b_1 |  | 7 | 2 | 5 | 6y, 15n | 0 | 0 | Y |  |
| 1b_2 |  | 7 | 1 | 6 |  |  |  |  |  |
| 1b_3 |  | 7 | 3 | 4 |  |  |  |  |  |
| 2b_1 |  | 7 | 0 | 7 | 0Y, 21N | 0 | 0 | Y |  |
| 2b_2 |  | 7 | 0 | 7 |  |  |  |  |  |
| 2b_3 |  | 7 | 0 | 7 |  |  |  |  |  |
| 3b_1 |  | 7 | 1 | 6 | 1Y, 20N | 0 | 1 | N | AI false pos |
| 3b_2 |  | 7 | 0 | 7 |  |  |  |  |  |
| 3b_3 |  | 7 | 0 | 7 |  |  |  |  |  |
| 4b_1 |  | 7 | 1 | 6 | 2Y, 19N | 0 | 0 | Y |  |
| 4b_2 |  | 7 | 1 | 6 |  |  |  |  |  |
| 4b_3 |  | 7 | 0 | 7 |  |  |  |  |  |
| 5b_1 |  | 7 | 1 | 6 | 1Y, 20N | 0 | 1 | N | AI false pos |
| 5b_2 |  | 7 | 0 | 7 |  |  |  |  |  |
| 5b_3 |  | 7 | 0 | 7 |  |  |  |  |  |
| 6b_1 |  | 7 | 0 | 7 | 0Y, 27N | 0 | 0 | Y |  |
| 6b_2 |  | 7 | 0 | 7 |  |  |  |  |  |
| 6b_3 |  | 7 | 0 | 7 |  |  |  |  |  |
| 6b_4 |  | 7 | 1 | 6 |  |  |  |  |  |
| 7b_1 |  | 7 | 0 | 7 | 0Y, 14N | 0 | 0 | Y |  |
| 7b_2 |  | 7 | 0 | 7 |  |  |  |  |  |
| 8b_1 |  | 7 | 2 | 6 | 18Y, 11N | 1 | 1 | Y |  |
| 8b_2 |  | 7 | 4 | 3 |  |  |  |  |  |
| 8b_3 |  | 7 | 6 | 1 |  |  |  |  |  |
| 8b_4 |  | 7 | 6 | 1 |  |  |  |  |  |
| 9b_1 |  | 7 | 2 | 5 | 3Y, 18N | 0 | 0 | Y |  |
| 9b_2 |  | 7 | 0 | 7 |  |  |  |  |  |
| 9b_3 |  | 7 | 1 | 6 |  |  |  |  |  |
| 10b_1 |  | 7 | 7 | 0 | 16Y, 5N | 1 | 1 | Y |  |
| 10b_2 |  | 7 | 7 | 0 |  |  |  |  |  |
| 10b_3 |  | 7 | 2 | 5 |  |  |  |  |  |
| 11b_1 |  | 7 | 1 | 6 | 1Y, 13N | 0 | 0 | Y |  |
| 11b_2 |  | 7 | 0 | 7 |  |  |  |  |  |
| 12b_1 |  | 7 | 0 | 7 | 2Y, 19N | 0 | 0 | Y |  |
| 12b_2 |  | 7 | 1 | 6 |  |  |  |  |  |
| 12b_3 |  | 7 | 1 | 6 |  |  |  |  |  |
| 1c_1 |  | 12 | 0 | 12 | 3Y, 45N | 0 | 0 | Y |  |
| 1c_2 |  | 12 | 0 | 12 |  |  |  |  |  |
| 1c_3 |  | 12 | 2 | 10 |  |  |  |  |  |
| 1c_4 |  | 12 | 1 | 11 |  |  |  |  |  |
| 2c_1 |  | 12 | 3 | 9 | 5Y, 31N | 0 | 0 | Y |  |
| 2c_2 |  | 12 | 1 | 11 |  |  |  |  |  |
| 2c_3 |  | 12 | 1 | 11 |  |  |  |  |  |
| 3c_1 |  | 12 | 2 | 10 | 4Y, 32N | 0 | 0 | Y |  |
| 3c_2 |  | 12 | 0 | 12 |  |  |  |  |  |
| 3c_3 |  | 12 | 2 | 10 |  |  |  |  |  |
| 4c_1 |  | 12 | 0 | 12 | 0Y, 48N | 0 | 0 | Y |  |
| 4c_2 |  | 12 | 0 | 12 |  |  |  |  |  |
| 4c_3 |  | 12 | 0 | 12 |  |  |  |  |  |
| 4c_4 |  | 12 | 0 | 12 |  |  |  |  |  |
| 5c_1 |  | 12 | 1 | 11 | 2Y, 33N | 0 | 0 | Y |  |
| 5c_2 |  | 12 | 1 | 11 |  |  |  |  |  |
| 5c_3 |  | 12 | 1 | 11 |  |  |  |  |  |
| 6c_1 |  | 12 | 9 | 3 | 14Y, 10N | 1 | 1 | Y |  |
| 6c_2 |  | 12 | 5 | 7 |  |  |  |  |  |
