# Supplementary material for: Reporting radiographers’ interaction with Artificial Intelligence—How do different forms of AI feedback impact trust and decision switching?
Source: PLOS Digit Health. 2024 Aug 7;3(8):e0000560. doi: 10.1371/journal.pdig.0000560 (PMC11305567; doi:10.1371/journal.pdig.0000560)
Supplement: Attachment — Submitted filename: Rebuttal tabulation May 2024 final.docx [file pdig.0000560.s003.docx]

**Rebuttal**

**Ulster University**

**School of Health Sciences/Computer Sciences**

**York Street**

**Belfast**

**16^th^ May 2024**

Dear Editor, Reviewers,

Thank you, most sincerely for consideration of this manuscript and the very helpful and valid comments. All suggested edits have been made and the changes are detailed below and as ‘tracked changes’ in the submitted document.

With kindest regards,

Clare

**Clare Rainey PhD FHEA**

**E:** [c.rainey@ulster.ac.uk](mailto:c.rainey@ulster.ac.uk) **W:** [www.ulster.ac.uk](http://www.ulster.ac.uk/)

**Social:** find me on LinkedIn and Twitter @ClareRaineyUU

**Orcid:** <https://orcid.org/0000-0003-0449-8646>

Manuscript reference: PDIG-D-24-00075

“Reporting radiographers’ interaction with Artificial Intelligence – how do different forms of AI feedback impact trust and decision switching”

Table 1: Journal requirements

| Please provide separate figure files in .tif or .eps format only and remove any figures embedded in your manuscript file. Please also ensure that all files are under our size limit of 10MB. | Done  Figure 3 is now suppl 1  Table 1 is now suppl 2  Figure 4 is now figure 3, 5 is now 4, 6 is now 5 and 7 is now 6 in both the figure labelling and the in text reference. |
| --- | --- |
| We do not publish any copyright or trademark symbols that usually accompany proprietary names, eg ©, ®, ™ (e.g. next to drug or reagent names). Please remove all instances of trademark/copyright symbols throughout the text, including © & ® on pages 2, 3, 8, 9 & 10. | Removed, thank you |

Table 2: Editor comments

| The reviewers commend the paper for its timely contribution to a pressing issue facing healthcare systems worldwide. However, they also point several limitations raised. While the study contributes valuable insights into trust dynamics, it's essential to acknowledge and address broader ethical and technical challenges. | Additionally, see rebuttal below.  Thank you. The authors have now addressed all comments in the feedback from the reviewers. Including the issue regarding the potential of human bias in interacting with AI. The reviewer note an additional paper and the authors have included this, however the paper by REF 37 (included in the original submission) is particularly relevant in this case: Gaube at al., in 2021 (Gaube S, Suresh H, Raue M, Merritt A, Berkowitz SJ, Lermer E, Coughlin JF, Guttag JV, Colak E, Ghassemi M. Do as AI say: susceptibility in deployment of clinical decision-aids. NPJ Digit Med. 2021 Feb 19;4(1):31. doi: 10.1038/s41746-021-00385-9. PMID: 33608629; PMCID: PMC7896064.) The suggested paper has now also been included in the manuscript, thank you.  *“Developers should be aware of this when creating forms of explainable AI and further study should be undertaken to investigate the impact of different forms of AI feedback on users’ trust, with particular cognisance of the potential insufficiencies in the use of current forms of explainable AI on the human end-user (Gaube at al., 2021; Jabbour et al., 2023).”* (line 605-607)  Reviewer #2 comments that the results may not be generalisable internationally. Some discussion on the limitations of the generalisability of the results to countries with low to middle income economies is now included. The authors have also reworded statement on page 3, regarding ‘global’ application to indicate that this may be generalisable to countries with high income economies and cultural background. The potential non-generalisability of the findings to countries with low and middle income economies has been noted.  The authors have also attempted to include this in the limitations section of the revised manuscript, although the various varied ethical frameworks and philosophical approaches to AI globally are not a focus of this work.  As this research did not gather data from other countries, the authors feel that it would not be responsible nor appropriate to speculate regarding the philosophical underpinnings and resultant behaviours of the trust construct across different countries. This reference has, again been included, however is a little out-of-scope for this paper.  *“The participants in this study were recruited from the UK only, which was intentional in an attempt to ensure that their role and educational background was similar. However, this may mean that the findings from this study may be generalisable to countries with high income economies and similar education and practice requirements only. Studies by Amugongo et al., 3032 and Ewuoso et al., 2023, note that the African (and, by extension potentially other countries with low to middle income economies) perspective on the concept of ‘trust’ may differ. Both studies suggest that, in this context, trust may be based on collective, experiential-based factors. This is beyond the scope of this study but should be considered in the context of potential disparities in cultural norms related to trust in AI and other emerging technologies.”* |
| --- | --- |
| The paper's generalizability is questioned due to the small sample size, lack of diversity among participants, and scope on musculoskeletal radiographs, which may not generalize across different areas of radiology. Long-term follow-up and comparison with human experts are suggested by reviewers. We look forward to addressing these comments on the revised manuscript. | This is addressed in the original manuscript and further clarity and rationale is now provided.  Thank you. As stated in the manuscript (line 283-286), this sample size has been chosen, based on recommendations in the literature for this type of study (Obuchowski, 2004; Allyn and Bacon, 2008). The authors agree that this is may be considered a small sample for some other study methodologies, however given methodology, focus and the novelty of this work, is felt to be well supported by the literature. However, this is now listed as a limitation in the manuscript, where recommendation for further research involving other health care/medical professionals involved in the interpretation of radiographic images for the provision of diagnosis:  *“Whilst this study recruited an adequate number of participants, as per current recommendation in the literature (Obuchowski, 2004; Allyn and Bacon, 2008) further research should be conducted with a larger number of participants with a varied background in the production of formal diagnosis from radiographic image interpretation, such as radiologists and other healthcare practitioners who provide formal written reports as part of their role.”*  This study intentionally recruited from the UK to control for the similarity in participant background. All participants were currently reporting radiographers and have received training and postgraduate education to the level required to practice in the UK. The scope of this paper was not to explore the correlations which may results from diversity in background and level of experience. This is partially explored in a related paper, produced by the same team and submitted along with this revision  Agreed, however, this study is focussing on the human interaction with forms of AI feedback and not the performance of a model on different anatomical areas. The team fully appreciate the complexity associated with the use of AI in different areas of radiology. As stated in the manuscript, the MURA dataset and images were chosen for this particular group and reporting in radiography is a specialised field, with the most common area of expertise being musculoskeletal radiography  This study is novel in this area and therefore it would not have been feasible to conduct further study at this point in time. AI implementation is still in its infancy in many places and therefore this type of pre-implementation research is important to try and pre-empt issues arising from clinical use. However, the authors agree that this is an important point of note for future studies and a much-required area of research to ensure other biases, such as automation complacency, do not begin to pervade with increased and prolonged clinical use. This has now been  included as a recommendation for further research:  *“Additionally, consideration should be given to long-term follow up of this study in this, and similar populations after a period of time using AI technologies in clinical practice to investigate any change in perception over time.”*  The participants in this study are classed as ‘experts’, due to their current clinical practice and subspecialist qualifications (within which interpretation of MSK images are a core element). This negates the need for additional input, as agreement between participants is deemed acceptable (line 215-217 and Section 4.2). Ground truth usually indicates agreement between usually 3 or more ‘experts’, as per similar: Rajpurkar P, Irvin J, Ball RL, Zhu K, Yang  B, Mehta H, et al. (2018) Deep learning for chest  radiograph diagnosis: A retrospective comparison  of the CheXNeXt algorithm to practicing  radiologists. PLoS Med 15(11): e1002686. https://  doi.org/10.1371/journal.pmed.1002686  *(see Table 4)* |
| *Staff editor note:* If you plan to revise or make a new submission of this work, please declare any related manuscripts in preparation or under consideration, describe the relationship between these works and provide a copy of the related work with your submission as a 'Related Manuscript' file type. Related works available as a preprint or accepted for publication at any time during the consideration/processing of a manuscript must be clearly cited and discussed. | Related manuscript (previously submitted to PlosDH) has been attached in its current form with this revised submission. Communication thread with Sarah Bangs in PLOS Publication Ethics team has also been copied verbatim below this table as well as in a further ‘cover letter’ submitted through the submission portal. |

*****

Dear Sarah,

Thank you so much. No problem at all. Thank you for your help.

Clare

Kindest Regards,

My workday may look different to your workday.  I am sending this e-mail now because it works for me. Please do not feel obligated to read, act on or respond outside of your normal working hours.

**Clare Rainey PhD FHEA**

Course Director BSc (Hons.) Diagnostic Radiography and Imaging

**T:** 028 71675980

**E:** [c.rainey@ulster.ac.uk](mailto:c.rainey@ulster.ac.uk) **W:** [www.ulster.ac.uk](http://www.ulster.ac.uk/)

**Social:** find me on LinkedIn and Twitter @ClareRaineyUU

**Orcid:** <https://orcid.org/0000-0003-0449-8646>

**From:** PLOS Pub Ethics <[pub-ethics@plos.org](mailto:pub-ethics@plos.org)>
**Sent:** 07 May 2024 13:20
**To:** Rainey, Clare <[c.rainey@ulster.ac.uk](mailto:c.rainey@ulster.ac.uk)>
**Cc:** McFadden, Sonyia <[s.mcfadden@ulster.ac.uk](mailto:s.mcfadden@ulster.ac.uk)>
**Subject:** RE: PLOS Digital Health: Information about related manuscripts policy

EXTERNAL EMAIL - This email was sent by a person from outside your organization. Exercise caution when clicking links, opening attachments or taking further action, before validating its authenticity.

[**Secured by Check Point**](https://www.checkpoint.com/harmony/email-security/email-office?friew)

Dear Dr Rainey,

Thank you for getting back to me and providing this additional context of how the two studies are related and how they differ. This is helpful, and I would like to share your message with the handling editor for PDIG-D-24-00075 if I may?

When you come to resubmit I would also ask that you include this in your cover letter alongside the copy of the related work (or if the related work is already accepted elsewhere at that point, discussion and citation should be included in the manuscript text).

Kind regards,

Sarah

Sarah Bangs
Senior Editor, Team Manager, Publication Ethics


PLOS
Empowering researchers to transform science


Public Library of Science
UK Registered Office Address: Nine Hills Road, Cambridge, CB2 1GE UK
Company registered in California, USA with UK Establishment Office in England and Wales
California (US) corporation #C2354500
UK Company # FC031758

CONFIDENTIAL: This email and any attachments are confidential and for the sole use of the individual(s) to whom they are addressed. If you have received this message in error please delete the message and notify the sender. Note that information and text in this correspondence should not be disclosed, copied, distributed, or used without explicit permission from PLOS.


--------------- Original Message ---------------
**From:** Rainey, Clare [c.rainey@ulster.ac.uk]
**Sent:** 07/05/2024, 12:03
**To:** [pub-ethics@plos.org](mailto:pub-ethics@plos.org)
**Cc:** [s.mcfadden@ulster.ac.uk](mailto:s.mcfadden@ulster.ac.uk)
**Subject:** RE: PLOS Digital Health: Information about related manuscripts policy

[CAUTION: External email. Proceed with Caution. ]

Dear Sarah,

Thank you very much for this.  I will absolutely submit the other paper with the updated publication.  We are not considering significant change to this when submitting to another publication.  These are distinct pieces of research, however, they were both undertaken as part of a PhD project, therefore the same dataset (MURA) used for training and testing (*only* training and testing as one of the studies used *clinical images* for the human interaction), the CNN type and the feedback used.  These were conducted with a very distinct type and population of participant and were aiming to discover very different human-computer interaction phenomena (trust impact with reporting radiographers using different feedback type and susceptibility of student v. radiographer automation bias/decision switching in relation to accuracy).  One study aims to uncover the impact of a poorly functioning AI (on the images provided – i.e., the clinical dataset) and the other uses an acceptably performing AI with no focus on accuracy (MURA images, with reporting radiographers providing ground truth themselves).  I’m afraid the papers would not be suitable for merging and, rather it was hoped that these papers would be published ‘together’ due to their relation in this niche field, and therefore provide readers with a holistic view of the facets of the human interaction with AI in clinical practice, using radiographers of differing levels of experience and professional focus as the example.  The team understand that the role and profession of radiography is varied internationally, and this may be leading to some confusion and perceived overlap.

Please also note, for accuracy, that the resubmission was due to a delay in journal review, rather than further research undertaken and the authorship hoped that both papers would be published in the same journal, hence the initial decision to withdraw until the second paper was ready for publication.

I am not sure if any of this helps, but I am very happy to discuss further.

Thank you very much again,

Kindest Regards,

*My workday may look different to your workday. ? I am sending this e-mail now because it works for me.  Please do not feel obligated to read, act on or respond outside of your normal working hours.*

**Clare Rainey PhD FHEA**

Course Director BSc (Hons.) Diagnostic Radiography and Imaging

**T:** 028 71675980

**E:** [c.rainey@ulster.ac.uk](mailto:c.rainey@ulster.ac.uk) **W:** [www.ulster.ac.uk](https://protect.checkpoint.com/v2/___http:/www.ulster.ac.uk/___.YzJlOnVsc3RlcnVuaXZlcnNpdHk6YzpvOjY1MTk2NzI0ZDhlNjk2YTg3NTBmYmU2MGRkOGJiNzJiOjY6NDFmNDpmMmIyNTRmNzJlYjQ5ZTVjMzJiNmNmN2RiNDU0Y2IwYWM1ZGRlNTI3NjdiNDBiMmZjNzIzNzY0NDYzYTZhYWFjOmg6VA)

**Social:** find me on LinkedIn and Twitter @ClareRaineyUU

**Orcid:** [https://orcid.org/0000-0003-0449-8646](https://protect.checkpoint.com/v2/___https:/orcid.org/0000-0003-0449-8646___.YzJlOnVsc3RlcnVuaXZlcnNpdHk6YzpvOjY1MTk2NzI0ZDhlNjk2YTg3NTBmYmU2MGRkOGJiNzJiOjY6YzRlNTo1OTY0OTVhZmY1NjE5NTBmMzIwZmY0ZDAzNmU4MTk1ZDU3ZTZhOTQ4YjgwZmM5ZTYyYWYwZDUxYzY4OTIyNWU5Omg6VA)

**From:** PLOS Pub Ethics <[pub-ethics@plos.org](mailto:pub-ethics@plos.org)>
**Sent:** Tuesday, May 7, 2024 11:21 AM
**To:** Rainey, Clare <[c.rainey@ulster.ac.uk](mailto:c.rainey@ulster.ac.uk)>
**Subject:** PLOS Digital Health: Information about related manuscripts policy

EXTERNAL EMAIL - This email was sent by a person from outside your organization. Exercise caution when clicking links, opening attachments or taking further action, before validating its authenticity.

[**Secured by Check Point**](https://protect.checkpoint.com/v2/___https:/www.checkpoint.com/harmony/email-security/email-office?friew___.YzJlOnVsc3RlcnVuaXZlcnNpdHk6YzpvOjY1MTk2NzI0ZDhlNjk2YTg3NTBmYmU2MGRkOGJiNzJiOjY6YWJjZDo5NzA0NTI0ZmQwMTBjYThjYjI0OTk1ODI1N2QxMDBjZmQ3MmE3ZWQ2MDk0MTdjMjNkNTA1ODBiZDM5Zjg1NTk1Omg6VA)

|  | You don't often get email from [pub-ethics@plos.org](mailto:pub-ethics@plos.org). [Learn why this is important](https://protect.checkpoint.com/v2/___https:/aka.ms/LearnAboutSenderIdentification___.YzJlOnVsc3RlcnVuaXZlcnNpdHk6YzpvOmZhNTM0Y2IzOGI1MTgxZWM2ZDU1ZTY5ZjlhOTNlMDM0OjY6MTMxYzpiYzhkOWVhYmNlNWZjNjM0MGM2YjA3NDQxM2JkZWY4NjljOGUwMTc0MzQ4ODhiOGRjZmIyYWE0YTIyY2ZlY2ZlOmg6VA) |  |
| --- | --- | --- |

Dear Dr Rainey,

I hope this message finds you well. I am writing in follow up to the recent editorial decisions on your submissions to PLOS Digital Health, both submitted on February 22 this year, and to provide some additional information regarding journal policy. The submissions in question are:

PDIG-D-24-00070 The impact of AI feedback on the accuracy of diagnosis, decision switching and trust in radiography.

PDIG-D-24-00075 Reporting radiographers’ interaction with Artificial Intelligence – how do different forms of AI feedback impact trust and decision switching

A concern was raised about similarity between these submissions. Upon preliminary assessment, we noted that the two manuscripts describe different studies, but that the research questions are closely related and partially overlapping, and the approach in each study uses the same MURA imaging dataset to train the same AI tool (ResNet-152 convolutional neural network (CNN)), and the same method for displaying the tool outputs as heatmaps, and that participants are asked to assess radiographic images, provide a diagnostic opinion, and self-rate whether this is affected by the AI tool’s feedback and their level of trust in the AI tool. It was noted that there were some differences in the specific task assigned to survey participants, in the range of experience of the participants, and in some of the analyses.   

I recognize that your cover letter for PDIG-D-100070 disclosed that you were resubmitting the manuscript having completed other research that you also hoped to publish in PLOS Digital Health. In view of the closely related nature of these two submissions, we consider both to fall under the journal’s policies for submission of related studies ([https://protect.checkpoint.com/v2/___https://journals.plos.org/digitalhealth/s/ethical-publishing-practice%23loc-submission-and-publication-of-related-studies___.YzJlOnVsc3RlcnVuaXZlcnNpdHk6YzpvOmZhNTM0Y2IzOGI1MTgxZWM2ZDU1ZTY5ZjlhOTNlMDM0OjY6Y2FiOTozOTE2ZWJmNTExMTE0YTdlMmM5MzBhNGQ0OTRjYTE3YWMyYzYyNzFjZGNlNmMzZDlhOTRjYjY5NzFlMGJhMjNhOnQ6VA](https://protect.checkpoint.com/v2/___https:/journals.plos.org/digitalhealth/s/ethical-publishing-practice%23loc-submission-and-publication-of-related-studies___.YzJlOnVsc3RlcnVuaXZlcnNpdHk6YzpvOmZhNTM0Y2IzOGI1MTgxZWM2ZDU1ZTY5ZjlhOTNlMDM0OjY6Y2FiOTozOTE2ZWJmNTExMTE0YTdlMmM5MzBhNGQ0OTRjYTE3YWMyYzYyNzFjZGNlNmMzZDlhOTRjYjY5NzFlMGJhMjNhOnQ6VA)), such that the editor and reviewers must be provided with a copy of the related manuscript under preparation/consideration (uploaded with the submission as a 'Related Manuscript' file type) and a description of how the studies are related to each other. This also applies to related works you may have submitted for review at other journals. This process allows for a fully informed editorial assessment of potential overlap in the research questions and whether the work can be considered independently (with appropriate citation and discussion). Related works available as a preprint or accepted for publication at any time during the consideration/processing of a manuscript must be clearly cited and discussed. 

At this time PDIG-D-100070 has already received a reject decision, while PDIG-D-00075 received a revise decision (with a staff editor note providing instructions about declaring and uploading related works). Please be aware that if you submit a revised manuscript for PDIG-D-100075, the editorial assessment will be carried out in light of the closely related work that is also concurrently under preparation by your group (which must be provided at submission); the editor/reviewers may advise after consideration of this information that PDIG-D-00075 is not suitable for independent reporting in PLOS Digital Health (and may potentially recommend combining the studies in a single manuscript). 

Please do not hesitate to get in touch if you have any questions about the above information and journal policies in this area. I would be grateful if you could reply by 10 May to confirm that you have received this message.

Kind regards,

Sarah

Sarah Bangs
Senior Editor, Team Manager, Publication Ethics


PLOS
Empowering researchers to transform science


Public Library of Science
UK Registered Office Address: Nine Hills Road, Cambridge, CB2 1GE UK
Company registered in California, USA with UK Establishment Office in England and Wales
California (US) corporation #C2354500
UK Company # FC031758

CONFIDENTIAL: This email and any attachments are confidential and for the sole use of the individual(s) to whom they are addressed. If you have received this message in error please delete the message and notify the sender. Note that information and text in this correspondence should not be disclosed, copied, distributed, or used without explicit permission from PLOS.

Case Number: 08461797
ref:!00DU00Ifis.!500PM0Bw3VU:ref
 

This email and any attachments are confidential and intended solely for the use of the addressee and may contain information which is covered by legal, professional or other privilege. If you have received this email in error please notify the system manager at [postmaster@ulster.ac.uk](mailto:postmaster@ulster.ac.uk) and delete this email immediately. Any views or opinions expressed are solely those of the author and do not necessarily represent those of Ulster University.
The University's computer systems may be monitored and communications carried out on them may be recorded to secure the effective operation of the system and for other lawful purposes. Ulster University does not guarantee that this email or any attachments are free from viruses or 100% secure. Unless expressly stated in the body of a separate attachment, the text of email is not intended to form a binding contract. Correspondence to and from the University may be subject to requests for disclosure by 3rd parties under relevant legislation.
**The Ulster University was founded by Royal Charter in 1984 and is registered with company number RC000726 and VAT registered number GB672390524.The primary contact address for Ulster University in Northern Ireland is Cromore Road, Coleraine, Co. Londonderry BT52 1SA**

Table 3: Reviewer #1 comments

| This is a timely and well-written manuscript that addresses a very important issue in healthcare delivery, not only for the UK, but quite broadly throughout the developed world where interpretive capacity of radiologists and non-radiologist interpreters of medical imaging is severely strained by burgeoning demand. The authors note that the development of AI has been viewed as a strategy for dealing with the need for substantial workflow augmentation in the NHS, coping with time-constraints and efficiency issues, as well as a strategy for error minimization. They cite the 2018 estimate that an effective AI system, if implemented effectively, could liberate nearly 1M hours of radiologist time per year. | Thank you. The authors very much appreciate the reviewer’s understanding of the focus of this work. |
| --- | --- |
| I was pleased with this paper overall, including its importance and rationale, clarity of the writing and presentation of the results, level of scientific rigor in study design and analysis, the quality of the analysis and discussion, the figures and tables, and the comprehensiveness of the reference list. | Sincere thanks |
| Although many research papers in this space are focused on evaluating the performance of AI algorithms in comparison to human observers, it is unlikely that such AI systems will ultimately be used to completely replace human beings in image interpretation--rather, they will be used to augment human performance and increase efficiency. As such, it is critical that the human-machine interface be optimized. One barrier for this optimization is trust, of course, which was the main focus of this manuscript. The systems must be adapted to optimize user trust. This is not unlike any other engineering problem. Also, optimal trust would include avoidance of both under-trust and over-trust. Another area where significant knowledge gaps remain is the ideal means for AI to provide its output to the human operators. | Thank you for this – the authors completely agree and have another paper under consideration currently, with an automation bias and decision switching focus (now included in this submission as related work). |
| This paper reports the results of a pilot study that addresses both of those issues. The authors focused on radiographic images of the appendicular skeleton which, in the UK, are primarily interpreted by non-radiologists. Interpreting radiographers are a skilled and experienced set of operators, but have less training and (by extension) likely less confidence than subspecialized musculoskeletal radiologists. For this reason, they are an ideal test-group for evaluating the human/AI interface, as it would be expected that these workers would benefit the most from AI augmentation. In the study reported, the trust placed by a small sample of interpreting radiographers in the AI algorithm was quantified both during and after AI outputs in the form of either a binary diagnosis (no explanation) or a heat / salience map on the radiograph (revealing what aspect of the image the AI conclusion was based on). The presumption embedded in the study is that trust will be proportional to the "explainability" of the AI output. While this core hypothesis of AI trust is eminently reasonable, this is something which nonetheless needs to be rigorously assessed. |  |
| The methods used in this project are sound. A standard image set (the MURA validation set) of ~3,200 images from 783 patients was used to train the AI, with a similar sized validation set from the same source. The AI system was trained to produce a salience or heat-map, in order to help the observer understand what the AI conclusion was based on, i.e., the areas of spatial location used by the model to determine the probability of a fracture. The study was carried out via email on the Qualtrics platform. A total of 12 participants were recruited, which seems a reasonable number (power calculations were not possible due to novelty). Inter-rater agreement was established using Kappa for two or more raters, a standard statistical method. Consensus diagnosis was considered to be "ground truth" and descriptive statistics were focused on determination of the level of agreement between AI and human observers, and compared to the ground truth. Correlation between trust and level of agreement was determined for the two types of AI output, as well as with image quality.  The results showed moderate agreement between participants with kappa = 0.581. This result is typical for radiology research comparing human interpreters. There was a fairly high level of agreement between the human operators with the AI, both in the binary feedback (86.7% agreement) and with the heat maps (54.1%, combining perfect and partial agreements). The participants indicated that they might switch their own decision to that of the AI, a measure of trust, in only 1.1% of the cases. The correlation analysis showed a large negative correlation between users' trust and the level of agreement with the heatmap, p > 0.01, but there were no significant correlations between trust and the likelihood that the observer would change their decision based on AI feedback. The latter result could be a sampling error due to the small number of participants, however in their discussion the authors note that decision-switching is also a factor to be optimized, and excessive decision-switching is to be avoided as much as an inappropriate resistance to decision-switching leading to fewer such switches than are needed to optimize accuracy. | Thank you. The authors agree that the results indicating a lack of correlation may be as a result of the smaller sample size, despite this number being accepted for studies of this type (Obuchowski, 2004; Allyn and Bacon, 2008). This has now been included as a limitation:  *“Whilst this study recruited an adequate number of participants, as per current recommendation in the literature (Obuchowski, 2004; Allyn and Bacon, 2008) further research should be conducted with a larger number of participants with a varied background in the production of formal diagnosis from radiographic image interpretation, such as radiologists and other healthcare practitioners who provide formal written reports as part of their role.”* |
| In this study, it would appear that the heat-map output was more effective than had been reported in previous studies, which the authors cite. There was a stronger correlation of users' trust with the heatmaps. Also, users indicated trust of the system when their decision matched the binary-output of the AI, which the authors correctly point out could be the result of human confirmation bias. In their discussion the authors also discussed the potential impact of the timing of AI feedback to the human observer on the ability of the AI to change the mind of the human user, both from the standpoint of avoiding anchoring bias and also from avoiding undue influence of the algorithm. The authors correctly point out that the "decision-switching" metric may differ between highly experienced, subspecialized clinician-users and novices or persons with less training and experience. In this cohort of participants, the likelihood of decision-switching was considered low.  The authors separately evaluated the effect of the diagnostic quality of the images, but this did not significantly effect the likelihood of a human observer to change their decision based on AI output. |  |
| The authors conclude that decision-switching may be independent of trust, and suggested that this could reflect anchoring bias by the human observers, but it could also reflect the low sample size. The authors call for future work with increased sample size and diversity, and suggest also that future studies should incorporate strategies to tease out the effect of anchoring bias. The authors are wise to suggest that future study should also include a broader range of formats for providing output to the clinician-user of the AI, in order to explore which formats are most effective at engendering trust, as well as making the AI feedback optional. One of the most interesting aspects of this work, from a human-factors standpoint, is that the AI-generated heat maps engendered greater trust in the algorithm, and yet the binary feedback (without explanation) was more preferred by the users! This result is remarkable and deserves further exploration. | Thank you – the authors are also very excited by this finding! Further research is ongoing within the authors’ team(s) and another relevant study from this team is now included with this submission. |
| The figures are excellent overall, especially Figs 3, 4, 6 and 7; Table 1 is a little tedious but is rewarding to the patient reader. Table 2 is very clear. Radiographic images are excellent and add to the readers' understanding. |  |
| Overall, as noted above, I found the paper to be solid in all relevant categories. I believe that it will generate substantial reader interest, stimulate future research, and should be well cited in the years ahead. The manuscript is well-composed and written, and so have no specific suggestions for changes or improvements. I commend the author group for a solid contribution to the literature, which is both timely and needed. I hope that others will take their challenge to extend this work in the future as they have suggested. | Most sincere thanks for this comment, and for all previous comments. We look forward to continuing our work in this area in the future and hope that it inspires further work from others. |

Table 4: Reviewer #2

| The paper investigates the impact of different forms of AI feedback on the trust and decision-making of reporting radiographers.  - The results show that participants had a greater level of agreement with the binary AI feedback than with the heat map localisation. Decision switching was low, with only two instances where participants indicated they would change their initial diagnosis based on the AI feedback.  - The study highlights the importance of developing explainable AI systems and considering the precision and accuracy of localisation to promote appropriate trust in clinical end users.  - The study highlights the importance of developing explainable AI systems and considering the precision and accuracy of localisation to promote appropriate trust in clinical end users. |  |
| --- | --- |
| However, XAI can also be biased because of bias data. A recent study has shown that clinicians can be fooled by bias AI systems, despite the system providing explanations for its outputs (jabour et al. 2023). Have the author noticed this?  Jabbour S, Fouhey D, Shepard S, et al. Measuring the Impact of AI in the Diagnosis of Hospitalized Patients: A Randomized Clinical Vignette Survey Study. JAMA. 2023;330(23):2275–2284. doi:10.1001/jama.2023.22295 | Thank you for this comment.  Yes, the authors are aware of this and also the paper by REF 37 Gaube at al., in 2021 (Gaube S, Suresh H, Raue M, Merritt A, Berkowitz SJ, Lermer E, Coughlin JF, Guttag JV, Colak E, Ghassemi M. Do as AI say: susceptibility in deployment of clinical decision-aids. NPJ Digit Med. 2021 Feb 19;4(1):31. doi: 10.1038/s41746-021-00385-9. PMID: 33608629; PMCID: PMC7896064.) The suggested paper has now also been included in the manuscript, thank you.  *“Developers should be aware of this when creating forms of explainable AI and further study should be undertaken to investigate the impact of different forms of AI feedback on users’ trust, with particular cognisance of the potential insufficiencies in the use of current forms of explainable AI on the human end-user (Gaube at al., 2021; Jabbour et al., 2023).”* (line 605-607) |
| The authors claim that though they  conducted their study in the UK, it can  be applied globally. This may not  necessarily true because trust is  complex in relational communities  Trust may be relational.  Lameck Mbangula Amugongo,  Nicola J. Bidwell, and Caitlin C. Corrigan.  2023. Invigorating Ubuntu Ethics in AI for  healthcare: Enabling equitable care. In  Proceedings of the 2023 ACM Conference  on Fairness, Accountability, and  Transparency (FAccT '23). Association  for Computing Machinery, New York,  NY, USA, 583–592. | Thank you for this good point. Some discussion on the limitations of the generalisability of the results to countries with low to middle income economies is now included. The authors have also reworded statement on page 3, regarding ‘global’ application to indicate that this may be generalisable to countries with high income economies and cultural background. The potential non-generalisability of the findings to countries with low and middle income economies has been noted.  The authors have also attempted to include this in the limitations section of the revised manuscript, although the various varied ethical frameworks and philosophical approaches to AI globally are not a focus of this work. |
| Also, as stated by Euwuso, “African view of trust as experience-based and accepting responsibility implies that health professionals can neither be held accountable for black box clinical AI outputs that they can hardly understand nor provide material information (concerning what the clinical AI does and why)”  Ewuoso, C. Black box problem and African views of trust. Humanit Soc Sci Commun 10, 705 (2023).  How does the authors findings reconcile with these views. | Point noted and addressed above in the limitations of this paper, which is not intended as a comparison between countries. As this research did not gather data from other countries, the authors feel that it would not be responsible nor appropriate to speculate. This reference has, again been included, however is a little out-of-scope for this paper.  *“The participants in this study were recruited from the UK only, which was intentional in an attempt to ensure that their role and educational background was similar. However, this may mean that the findings from this study may be generalisable to countries with high income economies and similar education and practice requirements only. Studies by Amugongo et al., 3032 and Ewuoso et al., 2023, note that the African (and, by extension potentially other countries with low to middle income economies) perspective on the concept of ‘trust’ may differ. Both studies suggest that, in this context, trust may be based on collective, experiential-based factors. This is beyond the scope of this study but should be considered in the context of potential disparities in cultural norms related to trust in AI and other emerging technologies.”* |
| The study has many limitations and the authors do not discuss this.  Sample size is one of them. How can one generalise with this sample size?  1. Small sample size: The study only included 12 participants, which may limit the generalizability of the findings. A larger sample size would provide more robust results. | Thank you. As stated in the manuscript (line 283-286), this sample size has been chosen, based on recommendations in the literature for this type of study (Obuchowski, 2004; Allyn and Bacon, 2008). The authors agree that this is may be considered a small sample for some other study methodologies, however given methodology, focus and the novelty of this work, is felt to be well supported by the literature. However, this is now listed as a limitation in the manuscript, where recommendation for further research involving other health care/medical professionals involved in the interpretation of radiographic images for the provision of diagnosis:  *“Whilst this study recruited an adequate number of participants, as per current recommendation in the literature (Obuchowski, 2004; Allyn and Bacon, 2008) further research should be conducted with a larger number of participants with a varied background in the production of formal diagnosis from radiographic image interpretation, such as radiologists and other healthcare practitioners who provide formal written reports as part of their role.”* |
| 2. Lack of diversity in participants: The study recruited reporting radiographers from four regions of the UK, but there may still be a lack of diversity in terms of demographics and experience. Explicitly mentioning the diversity of radiographers involved may help. | This study intentionally recruited from the UK to control for the similarity in participant background. All participants were currently reporting radiographers and have received training and postgraduate education to the level required to practice in the UK. The scope of this paper was not to explore the correlations which may results from diversity in background and level of experience. This is partially explored in a related paper, produced by the same team and submitted along with this revision. |
| 3. Limited scope of the dataset: The study focused on musculoskeletal radiographs of the upper extremity from the MURA dataset. This limited scope may not fully represent the challenges and complexities of AI interaction in other areas of radiology. | Agreed, however, this study is focussing on the human interaction with forms of AI feedback and not the performance of a model on different anatomical areas. The team fully appreciate the complexity associated with the use of AI in different areas of radiology. As stated in the manuscript, the MURA dataset and images were chosen for this particular group and reporting in radiography is a specialised field, with the most common area of expertise being musculoskeletal radiography. |
| 4. Lack of long-term follow-up: The study only assessed the participants' trust and decision switching immediately after exposure to AI feedback. It would be valuable to conduct long-term follow-up to assess the sustained impact of AI on trust and decision-making. Does the participants decision change if shown the same AI decision the next time. | This study is novel in this area and therefore it would not have been feasible to conduct further study at this point in time. AI implementation is still in its infancy in many places and therefore this type of pre-implementation research is important to try and pre-empt issues arising from clinical use. However, the authors agree that this is an important point of note for future studies and a much-required area of research to ensure other biases, such as automation complacency, do not begin to pervade with increased and prolonged clinical use. This has now been included as a recommendation for further research:  *“Additionally, consideration should be given to long-term follow up of this study in this, and similar populations after a period of time using AI technologies in clinical practice to investigate any change in perception over time.”* |
| 5. Lack of comparison with human performance: The study focused on the agreement between participants and AI feedback, but did not compare their performance to human experts. Comparing the performance of AI with human experts would provide valuable insights into the strengths and limitations of each approach. | The participants in this study are classed as ‘experts’, due to their current clinical practice and subspecialist qualifications (within which interpretation of MSK images are a core element). This negates the need for additional input, as agreement between participants is deemed acceptable (line 215-217 and Section 4.2). Ground truth usually indicates agreement between usually 3 or more ‘experts’, as per similar: Rajpurkar P, Irvin J, Ball RL, Zhu K, Yang  B, Mehta H, et al. (2018) Deep learning for chest  radiograph diagnosis: A retrospective comparison  of the CheXNeXt algorithm to practicing  radiologists. PLoS Med 15(11): e1002686. https://  doi.org/10.1371/journal.pmed.1002686 |
